# The Predictive Effects of Family and Individual Wellbeing on University Students' Online Learning During the COVID-19 Pandemic

**DOI:** 10.3389/fpsyg.2022.898171

**Published:** 2022-06-02

**Authors:** Xiaoqin Zhu, Carman K. M. Chu, Yee Ching Lam

**Affiliations:** Department of Applied Social Sciences, The Hong Kong Polytechnic University, Kowloon, Hong Kong SAR, China

**Keywords:** mediation, online learning, family support, life satisfaction, sleep difficulties

## Abstract

The COVID-19 pandemic has significantly changed university students' life routines, such as prolonged stay at home and learning online without prior preparation. Identifying factors influencing student online learning has become a great concern of educators and researchers. The present study aimed to investigate whether family wellbeing (i.e., family support and conflict) would significantly predict university students' online learning effectiveness indicated by engagement and gains. The mediational role of individual wellbeing such as life satisfaction and sleep difficulties was also tested. This study collected data from 511 undergraduate students (Mean age = 20.04 ± 1.79 years, 64.8% female students) *via* an online survey. Structural equation modeling analysis revealed positive effects of family support on students' learning engagement and gains through the mediational effects of life satisfaction and sleep difficulties. In contrast to our expectation, family conflict during the pandemic also positively predicted students' learning gains, which, however, was not mediated by individual wellbeing. The findings add value to the existing literature by delineating the inter-relationships between family wellbeing, individual wellbeing, and online learning effectiveness. The study also sheds light on the unique meaning of family conflict, which needs further clarification in future studies.

## Introduction

The outbreak and spread of the novel coronavirus disease (COVID-19) has led to lockdowns and social distancing measures worldwide. This has further resulted in “emergency remote teaching” such that educational institutions including universities moved onsite instructions online within a short period. For example, soon after the lockdown of Wuhan province in late January 2020, the Chinese Ministry of Education launched the initiative of “Disrupted Classes, Undisrupted Learning” in mid-February (Huang et al., 2020). This initiative provided flexible online learning for more than 270 million students. To prevent the rapid spread of the COVID-19 virus, the arrangement of online learning was extended to other places, such as the United States, the United Kingdom, India, South Korea, and Hong Kong (Baber, 2020; Burns et al., 2020; Cevasco et al., 2020).

Online learning is not a new pedagogy in higher education settings, and it has been increasingly popular in recent years. It is arguably due to online learning's advantages (e.g., flexible timetables, easy access to learning materials, reaching more students) and the advances in information technology (Bernard et al., 2014). However, unlike pre-pandemic online learning that is well-prepared and voluntarily adopted, “emergency remote teaching” as an alternative solution during the pandemic is “doing in a hurry with bare minimum resources and scant time” (Hodges et al., 2020). Therefore, institutions, teachers, and students have not got sufficient prior training and preparation for the overnight change. As such, students' online learning effectiveness during the pandemic is likely to be disrupted.

Learning effectiveness, which represents the quality of learning, is usually manifested in students' learning engagement and gains (Holzer et al., 2021; Tsang et al., 2021). Learning engagement refers to students' active participation in and commitment to learning-related tasks in terms of devoting time and efforts to completing academic activities that are key to desired outcomes (Reschly and Christenson, 2012; Yu et al., 2018). Learning gains, on the other hand, reflects educational success in form of how much students achieve in various aspects, such as academic achievement, skill improvement, and social development (Owston et al., 2013; Noesgaard and Ørngreen, 2015). Given the sudden change to online learning and insufficient preparation, researchers have raised concerns about students' online learning engagement and gains during the pandemic (Holzer et al., 2021; Tsang et al., 2021).

First, some students are not able to have a quiet physical environment at home for online studies (e.g., Aristovnik et al., 2020). This is typically the case in Hong Kong where several family members may live together in a small apartment while two or even more family members need to share one bedroom (Shek, 2021). Second, some university students have to compete for resources and equipment (e.g., computers, Wi-Fi networks, etc.) with other family members in supporting online learning (Shek, 2021). Third, some students may not adapt well to learning in a virtual environment due to a lack of digital devices, low technology literacy, poor time management skills, or low self-regulation. Their learning progress might fall behind. Fourth, students may not be able to obtain teachers' timely feedback and support or access to university resources when they encounter difficulties in learning. These speculations are supported by empirical findings. For example, Son et al. (2020) found that nearly 40% of the students felt difficulty in facing the sudden change and technical issues in online learning. The authors also reported that over 30% of the students worried about learning progress due to a lack of peer interactions and in-person teacher support.

Given difficulties or challenges students may encounter in learning during the COVID-19 pandemic, identifying factors that may promote or further impede students' online learning effectiveness becomes a great concern. Developmental systems theory maintains that individual development is a result of interactions between environmental systems (e.g., family, school, and society) and the individual (Lerner and Castellino, 2002). Among different systems, family plays an important role as it serves as the most immediate living environment, especially concerning the prolonged stay at home during the pandemic. According to family systems theory, family members reciprocally affect one another (Olson et al., 2019). Thus, family wellbeing that reflects the quality of family life, such as support, cohesion, or conflict between family members, is expected to affect students' individual life, including learning (Weng et al., 2015; Mangus et al., 2021).

Family support and family conflict can be considered two important measures of family wellbeing. When support from peers, teachers, or universities becomes less available under the pandemic, family support may be even more crucial in assisting students to be more self-regulated and resilient in online learning. For example, Permatasari et al. (2021) reported that family support made a greater contribution to students' academic resilience than did peer support and teacher support during the pandemic. Other two recent studies also showed that family support positively predicted students' learning effectiveness (e.g., engagement and motivation) during the pandemic (Koob et al., 2021; Yang et al., 2022).

However, the family could be a double-edged sword for students in the new learning mode. As afore-mentioned, family members may compete for family resources (e.g., space and equipment) when university students need to do online learning at home and other family members have to study or work from home as well. The prolonged stay at home may further intensify the contrasting views among family members and create more conflicts and stress (Husky et al., 2020). As a result, the two sides of family wellbeing during the pandemic may affect student learning differently. Family support motivates students and helps them better adapt to online learning. Conflict within the family, on the other hand, may create an unhealthy learning environment, possibly by intensifying anxiety and stress (Al-Kumaim et al., 2021). Indeed, while family support has been found to help college students release stress during the pandemic (Zhen et al., 2021), conflicts in the family exerted an additional burden on family members (Wu et al., 2020; Zainal Badri and Wan Mohd Yunus, 2022). Our proposition is also indirectly supported by Zeng et al.'s (2021) study. They found that high family cohesion (i.e., good relationships, communication, and few conflicts) reduced Chinese college students' stress responses to COVID-19. Nevertheless, direct evidence is limited, thus more research is needed to investigate whether family wellbeing would significantly influence university students' online learning effectiveness and in what ways.

One possibility is that family wellbeing may affect university students' online learning through the mediational effect of individual wellbeing. Individual wellbeing reflects the quality of one's personal life in physical (e.g., sleep quality) and non-physical domains (e.g., subjective wellbeing such as life satisfaction) (Shek, 2021). With good family wellbeing, such as having close emotional connectedness, open communication, and less conflict, individuals are more likely to be physically and mentally healthy. High family wellbeing (e.g., more communication and support but less conflict) has been empirically associated with greater life satisfaction and fewer physical and emotional problems among youths (Gunn and Eberhardt, 2019; Fosco and Lydon-Staley, 2020; Szcześniak and Tułecka, 2020). Similar findings have been reported during the pandemic such that higher family wellbeing was associated with better subjective and physical wellbeing among youngsters (Huang and Zhang, 2021; Zeng et al., 2021). It is possible that greater support and reliance from family members during hardship help individuals better deal with challenges and stress, allowing individuals to maintain a better status physically and psychologically.

Better individual wellbeing, in turn, acts as a favorable internal asset that facilitates students' online learning during the pandemic. According to the positive youth development framework, high individual wellbeing, which indicates one's well-functioning body, mind, and emotion, can be seen as a result of successful coping (Shek et al., 2019). Meanwhile, high individual wellbeing also predisposes the one to experience life events and deal with challenges and stress in a positive manner (Park, 2004). Rich findings have shown that university students are suffering from deteriorated wellbeing during the pandemic, such as physical illness, psychological distress, and dissatisfaction (Choi et al., 2020; Rogowska et al., 2020; Marelli et al., 2021). In this case, high individual wellbeing during the pandemic suggests students' overall effective adaptation during the crisis. Additionally, it also serves as a valuable personal resource that enhances students' further coping with demanding online learning requirements and tasks (Huang and Zhang, 2021; Koob et al., 2021). One measure of subjective wellbeing is life satisfaction which refers to an individual's cognitive assessment of the overall quality of life. High life satisfaction during the COVID-19 pandemic may predispose students to think and behave more positively and flexibly (Zhu and Shek, 2021), thus performing better in challenging online learning.

Although many studies have discussed university students' online learning, family wellbeing, and individual wellbeing during the pandemic, limited attention has been devoted to their inter-relationships in an integrated framework. In particular, no studies to date have tested the possible mediational role of individual wellbeing in linking family wellbeing and students' online learning effectiveness during the pandemic. The present study attempted to address this research gap by testing related hypotheses among university students in Hong Kong. We focused on two indicators of family wellbeing (family support and family conflict), two indicators of individual wellbeing (life satisfaction and sleep difficulties), and two measures of online learning effectiveness (learning engagement and learning gains). Based on previous elaborations, the present study had the following hypotheses.

**Figure 1:**
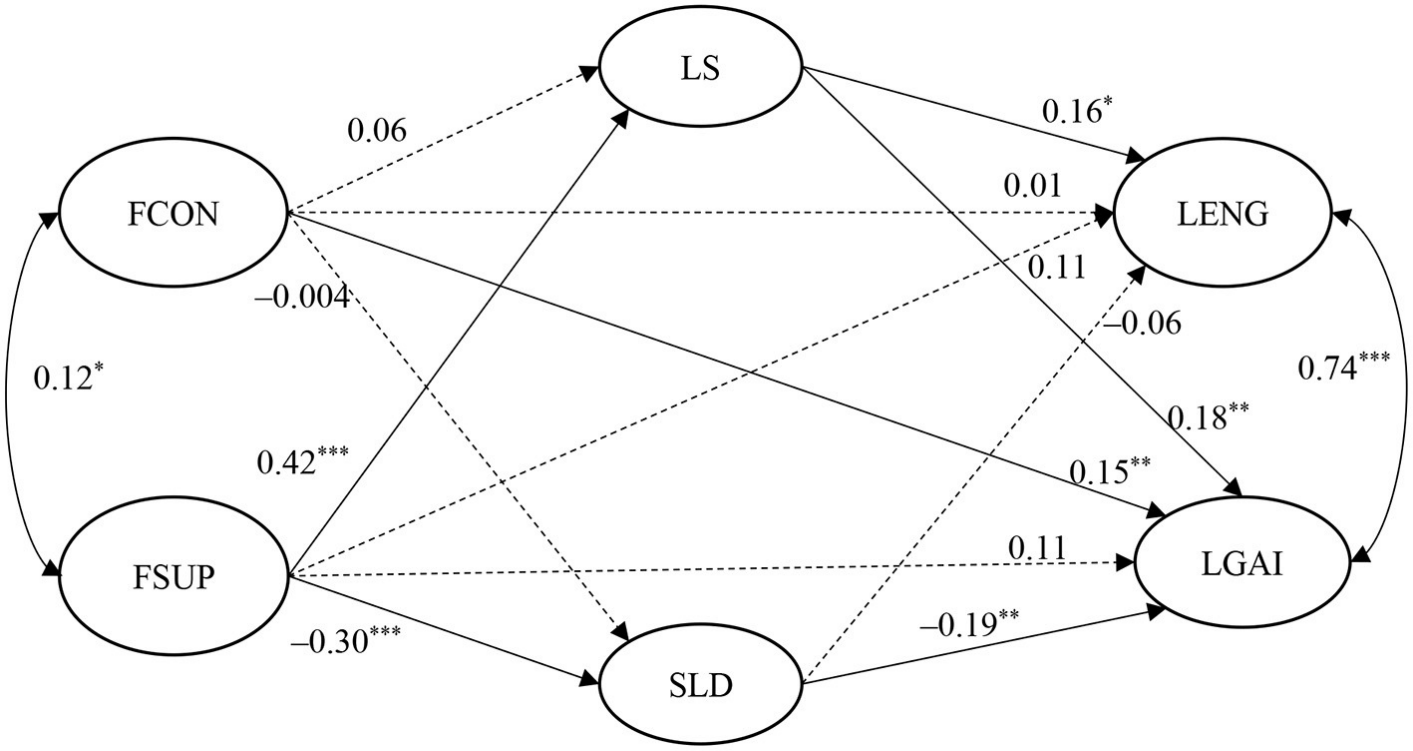
Standardized results of structural equation modeling on the relationships among family wellbeing, individual wellbeing, and learning (residuals and covariates are omitted for parsimony). FCON, family conflict; FSUP, family support; LS, life satisfaction; SLD, sleep difficulties; LENG, learning engagement; LGAI, learning gain. Solid lines indicate significant paths and dotted lines indicate insignificant paths. *p < 0.05, **p < 0.01, ***p < 0.001.

First, we expected that family support would positively predict learning engagement and gains (Hypotheses 1a and 1b) while family conflict would negatively predict learning engagement and gains (Hypotheses 1c and 1d). Second, we hypothesized that life satisfaction would positively predict learning engagement and gains (Hypotheses 2a and 2b) while sleep difficulties would negatively predict learning engagement and gains (Hypotheses 2c and 2d). Third, we expected that individual wellbeing measures would mediate the effect of family wellbeing on university students' online learning effectiveness (Hypothesis 3). A conceptual model encompassing these hypotheses is depicted in Figure 1 (it shows the results in terms of standardized path coefficients). We used a quantitative research design that collected data through an online survey. As such, data can be analyzed using advanced statistical techniques, such as structural equation modeling (SEM), to test the hypothesized associations between the constructs under investigation.

## Methods

### Participants and Procedures

In September 2021, a project entitled “Individual and family wellbeing among university students in Hong Kong under COVID-19” was launched at the authors' university in Hong Kong. This project aimed to understand undergraduate students' individual and family wellbeing and their relationships with student learning in the online teaching and learning environment during the pandemic. This project was reviewed and approved by the “Human Subjects Ethics Subcommittee” in the authors' university. The project collected data through an online survey from September to mid-October 2021 *via* a widely used platform *Qualtrics XM*. The survey link (and its associated QR code) was promoted by posts on the campus and emails sent by teachers teaching general education courses in the authors' department to their students. The participants were also encouraged to send the survey link to their classmates or friends at the university.

Students were invited to read an information sheet explaining the study purpose and essential principles upheld (e.g., confidentiality, voluntary participation, and free withdrawal) before giving their consent to participate in the survey. After giving their consent by clicking the button “I consent,” students were directed to the survey questions. Upon the completion of the survey, each participant received a HK$50 (roughly US$6.5) supermarket cash coupon as a token of appreciation (the participants were asked to provide an email address at the end of the survey for subsequent communication regarding coupon collection).

A total of 607 students in the university completed the survey. In online surveys, instructional manipulation check questions (e.g., “This is an attention check, please choose ‘strongly agree.”') are commonly employed to control data quality (Aust et al., 2013; Al-Salom and Miller, 2019). Following this practice, we also placed such a question in the survey where the respondents were instructed to select a specific answer. A total of 96 cases did not pass the check and they were excluded from the final analysis. As a result, the final effective sample consisted of 511 students (mean age = 20.04 ± 1.79 years, 64.8% female students). Among these participants, freshman students constituted the largest proportion (28.7%), followed by sophomores (26.4%), juniors (25.4%), and seniors (19.5%).

### Measures

#### Family Wellbeing

Two factors were assessed, including family support and family conflict during the pandemic. Family support was measured by the four-item “Family Support” subscale in the “Multidimensional Scale of Perceived Social Support” (Cheng and Chan, 2007). Participants rated the four items (e.g., “My family really tries to help me” and “I can talk about my problems with my family”) on a five-point scale from “1” (“strongly disagree”) to “5” (“strongly agree”). Family conflict was assessed by 10 items selected from the “Household Conflict” subscale in the “COVID-19 Family Environment Scale” (Behar-Zusman et al., 2020). These 10 items (e.g., “how to spend leisure time,” “home maintenance,” “finances,” “food,” and “privacy or personal space”) were deemed relevant to university students' life during the pandemic when they had to spend more time at home for online learning. Specifically, participants rated conflicts at home during social distancing as compared to the situation before COVID-19. A five-point scale was used (“1 = much fewer conflicts than before COVID-19, 5 = much more conflicts than before COVID-19”). In the present study, original items in the two scales were used. In other words, we did not make changes in measuring items. As shown in Table 1, the two scales measuring family wellbeing showed adequate reliability (Cronbach's αs and McDonald's ωs ranged between 0.76 and 0.89).

**Table 1:** Results of descriptive, reliability, and correlational analyses.

**Measure**	**Descriptions and reliability**					**Correlations**						
	**Mean**	**SD**	**Number of items**	**Average factor loading**	***α/ω***	**1**	**2**	**3**	**4**	**5**	**6**	**7**
1. Age	20.04	1.79				–						
2. Gender^a^						−0.13**	–					
3. Family conflict	3.35	0.55	10	0.50	0.76/0.76	−0.15**	0.14**	–				
4. Family support	3.50	0.86	4	0.80	0.88/0.89	−0.001	−0.06	0.12**	–			
5. Life satisfaction	3.46	0.96	5	0.77	0.87/0.87	−0.11*	0.12*	0.07	0.34***	–		
6. Sleep difficulties	1.56	0.80	10	0.61	0.86/0.86	0.01	0.02	−0.04	−0.26***	−0.22***	–	
7. Learning engagement	3.02	0.69	5	0.58	0.72/0.73	−0.04	−0.07	0.04	0.16***	0.18***	−0.07	–
8. Learning gain	3.39	0.64	7	0.67	0.85/0.85	0.04	−0.09	0.15**	0.23***	0.22***	−0.21***	0.59***

^a^1 = male, 2 = female. *p < 0.05; **p < 0.01; ***p < 0.001.

#### Individual Wellbeing

Life satisfaction and sleep difficulties, as indicators of subjective wellbeing and physical wellbeing, respectively, were measured in the present study. Life satisfaction was assessed by the five-item “Satisfaction with Life Scale” (SWLS), which has shown good reliability in literature (Zhu and Shek, 2020; Zhu et al., 2021). The participants gave their assessment on the five items (e.g., “In most ways, my life is close to my ideal.” and “The conditions of my life are excellent.”) on a six-point scale (“1 = strongly disagree, 6 = strongly agree”). Sleep difficulties were measured by the “Sleep Quality Questionnaire” developed and validated by Kato (2014). Among the 10 items, six were related to daytime sleepiness (e.g., “I sometimes felt sleepy during the day” and “I yawned frequently”) while the other four referred to difficulties in sleeping at night (e.g., “I had trouble sleeping” and “I felt like I did not get a deep sleep”). Students rated each item according to their own condition in the past one month on a five-point scale with “0” indicating “strongly disagree” and “4” indicating “strongly agree.” In this study, original items in the two scales were used. The two scales demonstrated adequate internal consistency as indicated by Cronbach's α values and McDonald's ω values (i.e., 0.86 and 0.87) shown in Table 1.

#### Learning Effectiveness

Learning effectiveness was indicated by learning engagement and learning gains. The two indicators were measured by the “Active Participation Scale” (APS) and the “Perceived Gain Scale” (PGS), respectively, which were developed and validated by Hsieh (2014). The five-item APS included behaviors such as raising questions, joining the in-class discussion, and working with classmates on the course project. The PGS included seven items. Four items referred to perceived personal and social gains (i.e., students' ability to learn and to understand themselves as well as to work effectively with others, four items). The other three items referred to perceived general educational growth (i.e., holistic academic development including thinking, writing, and speaking). We slightly revised the wording of these two scales (e.g., “in class” was changed to “in online class”) to make them suitable for measuring student online learning effectiveness. The participants were instructed to give their responses to the questions in APS and PGS on a five-point scale (“1 = not at all or never, 5 = very much or always”). In the present study, the two scales showed adequate internal consistency with Cronbach's α and McDonald's ω values varying between 0.72 and 0.85 (see Table 1).

### Data Analysis

Formal data analysis involved four steps, with SPSS 25.0 being used in the first step and Mplus 8.5 being used in other steps. First, we performed descriptive, reliability, and correlational analyses. Second, we checked the multivariate normality of measuring items on different scales. Results showed that the absolute values of skewness and kurtosis ranged between 0.10 and 1.17 (i.e., below two), indicating normal distributions of observed values. Therefore, the maximum likelihood (ML) estimation method can be properly utilized in subsequent SEM analyses (Finney and DiStefano, 2006).

Third, the measurement model of the conceptual model (Figure 1 depicts the model with standardized path coefficients) was tested through confirmatory factor analysis (CFA). In the model, there are six latent variables indicated by respective measuring items. Model fit indices, including CFI (“Comparative Fit Index”), TLI (“Tucker-Lewis Index”), RMSEA (“Root Mean Square Error of Approximation”), and SRMR (“Standardized Root Mean Square Residual”), were used to determine model fit. CFI and TLI values above 0.90 together with RMSEA and SRMR values below 0.08 indicate adequate model fit (Kline, 2015). CFA results revealed that the measurement model fitted the current data adequately: χ^2^ = 1,309.192, *df* = 759, χ^2^/*df* = 1.72, CFI = 0.917, TLI = 0.910, RMSEA = 0.038, SRMR = 0.053. In addition, the average factor loadings in each scale were above 0.50 (see Table 1). Thus, the structural model can be tested in the next step.

Fourth, the structural model shown in Figure 1 was tested with age and gender being statistically controlled. Bootstrapping with 1,000 times of re-sampling was employed to estimate the indirect effects of family wellbeing on students' learning. The same model fit indices and criteria used in CFA were adopted to determine model fit at this step.

## Results

The structural model well-fitted the data in the present study: χ^2^ = 1,440.678, *df* = 838, χ^2^/*df* = 1.72, CFI = 0.907, TLI = 0.900, RMSEA = 0.039, SRMR = 0.058. Figure 1 demonstrates the standardized path coefficients and Table 2 summaries direct and indirect effects with 95% confidence intervals.

**Table 2:** Standardized effects with confidence intervals in the conceptual model.

**Effects**	**Estimate**	**S.E**.	**95% CI**	
			**Lower**	**Upper**
**Total effects of family wellbeing on learning**				
Family conflict → Learning engagement	0.02	0.06	−0.09	0.15
Family conflict → Learning gains	0.16**	0.05	0.06	0.26
Family support → Learning engagement	0.20**	0.06	0.05	0.31
Family support → Learning gains	0.24***	0.06	0.14	0.35
**Direct effects of family wellbeing on learning**				
Family conflict → Learning engagement	0.01	0.06	−0.11	0.13
Family conflict → Learning gains	0.15**	0.05	0.05	0.24
Family support → Learning engagement	0.11	0.07	−0.03	0.24
Family support → Learning gains	0.11	0.06	−0.01	0.23
**Direct effects of family wellbeing on individual wellbeing**				
Family conflict → Life satisfaction	0.06	0.05	−0.04	0.17
Family conflict → Sleep difficulties	−0.004	0.06	−0.11	0.12
Family support → Life satisfaction	0.42***	0.05	0.31	0.52
Family support → Sleep difficulties	−0.30***	0.05	−0.40	−0.19
**Direct effects of individual wellbeing on learning**				
Life satisfaction → Learning engagement	0.16*	0.07	0.02	0.30
Life satisfaction → Learning gains	0.18**	0.06	0.06	0.30
Sleep difficulties → Learning engagement	−0.06	0.07	−0.20	0.08
Sleep difficulties → Learning gains	−0.19**	0.06	−0.30	−0.08
**Indirect effects of family wellbeing on learning through the mediation of individual wellbeing**				
Family conflict → Life satisfaction → Learning engagement	0.01	0.01	−0.003	0.04
Family conflict → Sleep difficulties → Learning engagement	0.000	0.01	−0.01	0.01
Family conflict → Life satisfaction → Learning gains	0.01	0.01	−0.01	0.04
Family conflict → Sleep difficulties → Learning gains	0.001	0.01	−0.03	0.02
Family support → Life satisfaction → Learning engagement	0.07*	0.03	0.01	0.13
Family support → Sleep difficulties → Learning engagement	0.02	0.02	−0.02	0.06
Family support → Life satisfaction → Learning gains	0.07**	0.03	0.03	0.14
Family support → Sleep difficulties → Learning gains	0.06**	0.02	0.02	0.10

CI, confidence interval. *p < 0.05; **p < 0.01; ***p < 0.001.

First, family support showed an overall significant positive prediction on both learning engagement (β = 0.20, *p* < 0.01) and learning gains (β = 0.24, *p* < 0.001) in an online learning environment. Family conflict during the COVID-19 pandemic showed an overall significant positive effect on perceived learning gains (β = 0.16, *p* < 0.01) while its effect on learning engagement was not significant. Thus, Hypotheses 1a and 1b were supported while Hypotheses 1c and 1d were not.

Second, as far as individual wellbeing indicators were concerned, life satisfaction acted as a positive predictor of both learning engagement (β = 0.16, *p* < 0.05) and gains (β = 0.18, *p* < 0.01) whereas sleep difficulties only negatively predicted learning gains (β = −0.19, *p* < 0.01). These findings fully supported Hypotheses 2a, 2b, and 2d (i.e., Hypothesis 2c was not supported).

Third, regarding the mediational effect of individual wellbeing, the effect of family support on learning engagement was mediated by life satisfaction (β = 0.07, *p* < 0.05), but not sleep difficulties (β = 0.02, *p* > 0.05). The effect of family support on learning gains was mediated by both life satisfaction (β = 0.07, *p* < 0.01) and sleep difficulties (β = 0.06, *p* < 0.01). In contrast, neither life satisfaction nor sleep difficulties mediated the prediction of family conflict on students' learning effectiveness. The reason is that family conflict during the COVID-19 pandemic did not significantly predict the two individual wellbeing measures. As a result, the effect of family conflict on learning gains was attributed to the direct effect (β = 0.15, *p* < 0.01). Thus, Hypothesis 3 was partially supported by the present findings.

## Discussion

Since early 2020, the COVID-19 pandemic has forced a sudden transition from face-to-face teaching and learning to online education in universities worldwide, including the authors' university in Hong Kong. Such a change is distinct from planned online learning because students, teachers, and institutions do not have sufficient time to adapt to the “one-night” change. Thus, the sudden move to online learning may impede student learning effectiveness. In addition, due to its uncertain consequences and associated social distancing measures, the COVID-19 pandemic may negatively affect students' individual wellbeing (e.g., physical and mental health) and family wellbeing (e.g., support and conflict) (Pappa et al., 2020; Wang et al., 2020; Shek, 2021). Previous studies have separately investigated students' online learning, their individual wellbeing, and family wellbeing during the COVID-19 pandemic. The present study expanded the previous research scope by testing how family and individual wellbeing would impact university students' online learning effectiveness using a mediational model.

Overall speaking, family support showed positive effects on students' learning effectiveness in terms of online learning engagement and perceived learning gains. This is consistent with prior findings showing positive impacts of family support on students' e-learning engagement during the pandemic (Domina et al., 2021; Gao et al., 2021). During the pandemic, family tended to play a major role in students' life because direct peer support and university support became less accessible and influential (Tsang et al., 2021). Together with prior research, our findings imply that family support may serve as a significant external resource that helps students better adapt to the new learning pedagogy. In particular, when students have to learn online at home, family support, in terms of emotional support, discussion on difficulties, and willingness and readiness to offer help, is likely to create a nurturing learning environment. Such a harmonious environment is conducive to the development of learning competence and motivation that make students more resilient in coping with learning difficulties during online learning (Gao et al., 2021; Mo et al., 2021). Indeed, Mo et al. (2021) reported that students perceiving more family support found it easier to use online learning during the pandemic.

Our findings suggest that the positive effects of family support on student learning during the COVID-19 pandemic operated through the meditational effects of individual wellbeing. This observation supports the developmental systems theory that individual development is an interaction of external and internal factors (Lerner and Castellino, 2002). Previous studies have also supported the mediational role of internal assets (e.g., psychological wellbeing, competence, and self-efficacy) in linking external assets (e.g., family) and individual developmental outcomes (Gao et al., 2021; Shek et al., 2022). Primarily, university students have been found to suffer from poor physical health (i.e., sleep disturbance) and subjective wellbeing (i.e., low life satisfaction) during the pandemic due to feelings of uncertainty, fear, anxiety, and hopelessness (Xiong et al., 2020; Duong, 2021). Our findings suggest that family support may help buffer these negative consequences by providing individuals with essential external resources to overcome psychological crises under stress. This conjecture is in line with some previous research findings. For example, Zeng et al. (2021) reported that family cohesion characterized by friendly relationships among family members negatively predicted fear of COVID-19 and stress consequences (e.g., insomnia).

Meanwhile, students' individual wellbeing was significantly associated with their learning effectiveness, with higher life satisfaction and fewer sleep difficulties predicting greater learning effectiveness. This observation echoes a previous conclusion that one of the obstacles students encountered in online learning is personal health challenges (Al-Kumaim et al., 2021). Previous studies have observed positive associations between life satisfaction and students' learning engagement and achievement (e.g., Lewis et al., 2011; Heffner and Antaramian, 2016). According to some scholars (Fredrickson, 2001; Park, 2004), high life satisfaction represents intellectual and emotional flexibility and recourses that enable individuals to experience life and cope with stress (e.g., academic stress) more effectively. In the current case, university students with higher life satisfaction may interpret and adapt to online learning (and other changes) more positively and thus have higher learning effectiveness under the “new normal.”

In addition, fewer sleep difficulties imply less stress and sufficient rest that let students better revitalize and restore their physiological processes. This, in turn, helps keep students' bodies and minds functioning efficiently in online learning (e.g., staying attentive without daytime sleepiness), leading to greater learning gains (Pascoe et al., 2020). However, the present study did not identify a significant relationship between sleep difficulties and online learning engagement. This finding is different from previous observations showing that sleep quality was positively associated with student learning engagement (Ng et al., 2022). One possible explanation is that students with better body and mind functioning may not necessarily be more engaged in asking questions or discussing with others. Instead, they may be more engaged in terms of individual thinking, comprehending, and reflecting, which was not assessed in the present study. Future studies will benefit from distinguishing between different forms of learning engagement (e.g., cognitive, emotional, and behavioral engagement).

In contrast to our hypothesis, family conflict during the COVID-19 pandemic did not exert negative effects on students' learning effectiveness. Instead, it showed an overall direct positive impact on perceived learning gains while not significantly predicting students' life satisfaction or sleep difficulties. One possible explanation is that having “family conflict” may not indicate that “family is not supportive,” which is believed to cause extra stress and challenges to students' online studies during the pandemic (Al-Kumaim et al., 2021). Instead, family conflict may emerge from frequent communication amongst family members, and despite the presence of disagreement, family members still support each other. This speculation is evidenced by the positive correlation between family support and family conflict in the present study. This is also in line with Shek (2021) notion that prolonged stay at home during the pandemic promotes communication and family cohesion while simultaneously creating more conflicts. Likely, family conflict per se did not represent an unfavorable family environment that hampers students' wellbeing and online learning. What really matters might be how family members resolve conflicts and provide mutual support. Given the present finding is novel, more studies are needed to further investigate how family support and conflict may affect student wellbeing and learning under stress.

The above-mentioned interpretations of the present findings should be considered with the notice of several limitations of this research. First, this is a cross-sectional study. Thus, the associations between family wellbeing, individual wellbeing, and student learning effectiveness cannot establish causality. Reciprocal relationships may exist among the three constructs. For example, learning effectiveness may also contribute to life satisfaction, or students with higher life satisfaction may tend to perceive more family support. Future studies need to collect longitudinal data to test these possibilities. Second, this study only recruited students from one university. Diversified samples should be employed in future studies to enhance the generalizability of the research findings. Third, the present study did not take into consideration the influence of institutional factors (e.g., teacher support and peer interaction), which should be examined and compared with family factors in future studies.

Despite these limitations, this study adds value to the existing literature by investigating the contribution of family and individual wellbeing to student learning effectiveness in an online learning context during the pandemic. The significant paths from family support to life satisfaction and sleep quality and in turn to learning engagement and gains revealed the significant mediational roles of individual wellbeing. Family support significantly and positively predicted online learning effectiveness and such an effect was fully mediated by life satisfaction and sleep quality. These results can be interpreted under the framework of developmental system theory and imply the importance of enhancing both family and personal wellbeing during a crisis. Family members, especially parents, are recommended to create a healthy and harmonious household environment that is conducive to students' wellbeing and learning. Meanwhile, university students are suggested to better communicate with parents to purposefully maintain a nurturing family environment in which they can keep better subjective and physical wellbeing and maximize learning effectiveness.

## Data Availability Statement

The raw data supporting the conclusions of this article will be made available by the authors, without undue reservation.

## Ethics Statement

The studies involving human participants were reviewed and approved by Human Subjects Ethics Sub-Committee at the Hong Kong Polytechnic University. The patients/participants provided their written informed consent to participate in this study.

## Author Contributions

XZ designed the project, interpreted the data, drafted, and finalized the work. CC contributed to data collection and drafted the work. YL contributed to the design of the project and data collection. All authors contributed to the article and approved the submitted version.

## Funding

This work was supported by the Undergraduate Research and Innovation Scheme (URIS) granted to XZ and YL (Project number: P0038339) and a start-up grant to XZ (Project number: P0034745) at The Hong Kong Polytechnic University.

## Conflict of Interest

The authors declare that the research was conducted in the absence of any commercial or financial relationships that could be construed as a potential conflict of interest.

## Publisher's Note

All claims expressed in this article are solely those of the authors and do not necessarily represent those of their affiliated organizations, or those of the publisher, the editors and the reviewers. Any product that may be evaluated in this article, or claim that may be made by its manufacturer, is not guaranteed or endorsed by the publisher.
